# Development of a counterselectable seamless mutagenesis system in lactic acid bacteria

**DOI:** 10.1186/s12934-017-0731-8

**Published:** 2017-07-05

**Authors:** Yongping Xin, Tingting Guo, Yingli Mu, Jian Kong

**Affiliations:** State Key Laboratory of Microbial Technology, Shandong University, 27 Shanda Nanlu, Jinan, 250100 People’s Republic of China

**Keywords:** Lactic acid bacteria, Temperature-sensitive plasmid, Seamless mutagenesis, Counterselectable marker, *pheS*

## Abstract

**Background:**

Lactic acid bacteria (LAB) are receiving more attention to act as cell factories for the production of high-value metabolites. However, the molecular tools for genetic modifying these strains are mainly vector-based double-crossover strategies, which are laborious and inefficient. To address this problem, several counterselectable markers have been developed, while few of them could be used in the wild-type host cells without pretreatment.

**Results:**

The *pheS* gene encoding phenylalanyl-tRNA synthetase alpha subunit was identified in *Lactococcus lactis* NZ9000 genome. When mutant *pheS* gene (pheS*) under the control of the *Lc. lactis* NZ9000 l-lactate dehydrogenase promoter (P_ldh_) was expressed from a plasmid, the resulted PheS* with an A312G substitution rendered cells sensitive to the phenylalanine analog *p*-chloro-phenylalanine (*p*-Cl-Phe). This result suggested pheS* was suitable to be used as a counterselectable marker in *Lc. lactis*. However, the expression level of pheS* from a chromosomal copy was too low to confer *p*-Cl-Phe sensitivity. Therefore, a strategy of cascading promoters was attempted for strengthening the expression level of pheS*. Expectedly, a cassette 5Pldh-pheS* with five tandem repetitive promoters P_ldh_ resulted in a sensitivity to 15 mM *p*-Cl-Phe. Subsequently, a counterselectable seamless mutagenesis system PheS*/pG^+^host9 based on a temperature-sensitive plasmid pG^+^host9 harboring a 5Pldh-pheS* cassette was developed in *Lc. lactis*. We also demonstrated the possibility of applying pheS* to be a counterselectable marker in *Lactobacillus casei* BL23.

**Conclusions:**

As reported in *E. coli*, pheS* as a counterselectable marker has been demonstrated to be functional in targeted gene(s) deletion in *Lc. lactis* as well as in *L. casei*. Moreover, the efficiency and timesaving counterselectable seamless mutagenesis system PheS*/pG^+^host9 could be used in the wild-type host cells without pretreatment.

## Background

Lactic acid bacteria (LAB) are important microorganisms used as starter cultures in the dairy fermented processes [1, 2]. Due to their generally recognized as safe status, some LAB strains have been used as cell factories or vaccine delivery vehicles for the heterogeneous production of specific compounds [3, 4] or pharmaceutical molecules [5–8]. Also, since the wealth of genomic data being delivered by massively parallel sequencing, interests in development of high-efficiency genome engineering tools for rerouting natural metabolic pathways to produce high valuable end products were increasing [9, 10]. Considering of the importance of the recombineering system in LAB, significant efforts have been recently concentrated on the exploitation of gene targeting techniques in LAB to accelerate genome engineering or gene functional analysis, as the recently reported single-stranded DNA recombineering (SSDR) system and double-stranded DNA recombineering (DSDR) system [11–13].

The SSDR system was established in *Lactobacillus reuteri* and *Lactococcus lactis* and could generate precision genome mutations without leaving any other foreign DNA [11]. The major limitation is the ability to achieve efficiencies that would allow the modification of any sites in the genome and easily recover the mutants without selection [14]. To address this problem, enhanced SSDR has been achieved with the assistant of the type-II clustered regularly interspaced short palindromic repeats locus from *Streptococcus pyogenes* in *L. reuteri* and >99.99% of non-recombinants could be eliminated without antibiotic selection [12]. But it could not be used for gene(s) deletion or insertion in other LAB because it has not proved the functional application of type-II CRISPR–Cas system in LAB except for *L. reuteri*. The DSDR technique was established in *L. plantarum* which was involved in the efficiently generation of gene(s) deletion or insertion [13]. However, this genetic system was not functional in other LAB and still left a *lox72* site, a heterologous DNA sequence after the antibiotic selection marker excised from the genome.

Seamless mutagenesis refers to targeted mutagenesis without any other micro-change, such as the presence of the selectable marker used to screen mutants or a *loxP* site after excising the selectable marker [15]. The seamless mutagenesis strategy usually appropriate for mutating the protein coding region in which any extraneous sequence introduced could interfere with protein expression. So far, several seamless mutagenesis methods based on homologous double-crossover have been successfully achieved in LAB, but the most widely used seamless mutagenesis strategy was based on a temperature-sensitive plasmid such as pG^+^host9 [16] or pG^+^host5 [17]. The merit of these plasmids is that both the non-replicate temperature at 37 °C and the replicate temperature at 28 °C are the adaptive temperature for the growth of most LAB. With plasmid pG^+^host9 [16], several chromosomal deletion derivatives of *Lc. lactis* and *Streptococcus thermophilus* were obtained in our laboratory [18–20]. However, these vectors, while powerful, suffer from a relatively low rate of recombination events and require labor-intensive screening procedures to distinguish clones with the desired seamless mutants. Therefore, improving the efficiency of this seamless mutagenesis system for fast analysis the function of gene(s) in LAB is very instant.

In recent years, a two-step selection/counterselection strategy has been demonstrated to be functional in improving the efficiency of method for fast generating seamless mutagenesis in the genome, which is normally consist of a positive selectable marker (usually an antibiotic resistance gene) and a counterselectable cassette. Counterselectable markers, including the genes *upp* [21–23] and *oroP* [24], have been characterized and functionally analyzed. However, the counterselectable marker *upp* could not be made in wild-type LAB strains without pre-treatment while *oroP* has not been widely used for other LAB strains [21–24].

Recently, gene *pheS* encoding phenylalanyl-tRNA synthetase alpha subunit has been demonstrated to function as a host strain-independent counterselectable marker in *Thermus thermophilus*, *Bacteroides* sp., *Escherichia coli* and *Streptococcus mutans* [25–28], but has yet not been used in the model strain *Lc. lactis*. In *E. coli*, only an A294G substitution in the protein PheS altered the specificity of the phenylalanyl-tRNA synthetase which resulted in the sensitivity to phenylalanine analogs such as *p*-chloro-phenylalanine (*p*-Cl-Phe) [27]. In this study, we identified a conserved alanine residue in the PheS protein, and demonstrated that the dominant-negative mutant protein PheS* with an A312G amino acid substitution rendered cells sensitive to 15 mM *p*-Cl-Phe in *Lc. lactis* NZ9000 and 10 mM *p*-Cl-Phe in *L. casei* BL23. To employ this conditional lethal gene pheS* as a negative selectable marker, a high-efficiency seamless mutagenesis system PheS*/pG^+^host9 based on a temperature-sensitive plasmid pG^+^host9 carrying a 5P_ldh_-pheS* cassette was constructed in *Lc. lactis* NZ9000. The aim of this study is to explore the potential of using pheS* as a counterselectable marker for rapidly screening mutants for targeted gene analysis or genome engineering in LAB.

## Methods

### Plasmids, bacterial strains, and growth conditions

The plasmids and bacterial strains used in this study are shown in Table 1. *E. coli* DH5α was used as the host for cloning procedures and grown aerobically in Luria–Bertani (LB) medium at 37 °C. Unless otherwise specified, *Lc. lactis* and *L. casei* were grown statically at 30 °C in M17 (Oxoid) broth supplemented with 0.5% glucose (GM17) and at 37 °C in MRS (Oxiod) broth, respectively. For counterselection, semi-defined M9 plates [29] supplemented with 0.4% glucose, namely GM9 plates, were added with 15 mM *p*-Cl-Phe (Sigma) for *Lc. lactis* and 10 mM *p*-Cl-Phe (Sigma) for *L. casei*. If required, antibiotics were added as follows: 10 µg/ml erythromycin or 5 µg/ml chloramphenicol for *Lc. lactis*, 5 µg/ml erythromycin for *L. casei*, 10 µg/ml chloramphenicol, 100 µg/ml ampicillin and 300 µg/ml erythromycin for *E. coli* DH5α.

**Table 1:** Plasmids and bacterial strains used in this study

Strain or plasmid	Characteristic(s)	Source
Strains		
*Escherichia coli* DH5α	F^−^ *supE44 ∆lacU169* Ф80*lacZ ∆M15 hsdR17 recA1 endA1 gyrA96 thi*-*1 relA1*	Novagen
*Lactococcus lactis* strains		
NZ9000	Derivative of MG1363, *pepN*::*nisRK*	[33]
IGn	Derivative of NZ9000, *galK*::pG^+^UD-nP-pheS*	This work
dA	Derivative of NZ9000, *∆aldB*	This work
*Lactobacillus casei* BL23	Derivative of *L. casei* ATCC 393 (pLZ15^−^)	[36]
Plasmids		
pG^+^host9	Erm^r^; temperature-sensitive vector	[16]
pG^+^UD	pG^+^host9 derivative with up and down homology arms of the part of galactose operon	This work
pG^+^UD-nP-pheS*	pG^+^UD derivative with pheS* driven by n P_ldh_	This work
pG^+^-nP-pheS*	pG^+^host9 derivative with pheS* driven by n P_ldh_	This work
pG^+^UD2-5P-pheS*	pG^+^-5P-pheS* derivative with upstream and downstream sequences of the *aldB* gene	This work
pUC19	Amp^r^; cloing vector	This work
pSec:Leiss:Nuc	pWV01 replicon; expresses Nuc under PnisA control; Cm^r^	[34]
pleiss-P-pheS*	pSec:Leiss:Nuc derivative with pheS* driven by a P_ldh_	This work
pTRKH2	Erm^r^; expressing vector	[37]
pOgfp	Source of *gfp* gene	[18]

### Reagents and enzymes

All enzymes used in this study were purchased from TaKaRa. Restriction enzymes and T4 DNA ligases were used according to standard procedures. PCR amplicons for cloning purposes were generated by 2× PrimeSTAR max premix, and PCR reactions for screening purposes were performed with rTaq DNA polymerase. All oligonucleotides used in this study are listed in Table 2.

**Table 2:** Oligonucleotide primers used in this study

Primer	Sequence (5′–3′)^a^	Restriction site
aldB-uF	AGGGTACCGGCGAAAGTCATGTAACAATCC	*Kpn*I
aldB-uR	CTGACATGATATTTCTCTTTTCTAT	
aldB-dR	CCGCTCGAGTGCTGACAGATGGCTGGCTGTG	*Xho*I
aldB-dF	GAAAAGAGAAATATCATGTCAGTAATTGCTTAAATTTCTTTAGC	
aldB-testF	ATATTTCTGCCACAATTTTCATGCC	
aldB-testR	CCAATCCTGTACCAATAACAGCAAT	
pheSF	ATAAAAAATCGAAAAGGAGATAAAAATGAACTTACAAGAAAAAATTGAAG	
pheSR	AAAGATCTTCAGTCGAATTGTTCTAAGAATC	*Bgl*II
pheSR2	AAGAATTCTCAGTCGAATTGTTCTAAGAATC	*Eco*RI
siteR	ACCAAAACCAGAATAAACAGAAG	
siteF	CTGTTTATTCTGGTTTTGGTTTTGGACTCGGTCAAGAACG	
ldhF1	CCGAATTCATTCATTTTACACATTGTA	*Eco*RI
ldhF2	GACAGATCTATTCATTTTACACATTGTA	*Bgl*II
ldhF3	AGACGTCGACATTCATTTTACACATTGTA	*Sal*I
ldhR1	TTTTATCTCCTTTTCGATTTTTTAT	
ldhR2	CGGGATCCTTTTATCTCCTTTTCGATTTTTTAT	*Bam*HI
ldhR3	CCGCTCGAGTTTTATCTCCTTTTCGATTTTTTAT	*Xho*I
upF	AGGGTACCATGTCAATAGTTGTCGAAAA	*Kpn*I
upR	CAGTTTCTGCTAAGGTATCA	
downF	TGATACCTTAGCAGAAACTGATGAATTAGCACAGCAAGTG	
downR	CCGCTCGAGCTCTAGTAAAATGTTCCTCA	*Xho*I
testF	TTAAGGAAATGAATTTAGAGGAGAG	
testR	AAACCTTCATGTCCTTCTTGAGT	
BL-pheSF	AAAGATCTATGGATCTTCAAACCAAACTTG	*Bgl*II
BL-siteR	ACCAAAACCGCCGTAAACGTC	
BL-siteF	GACGTTTACGGCGGTTTTGGTTTTGGCCTTGGTCCTGATCG	
BL-pheSR	GATCTGCAGTTAACCCTCCTGGCTGAATTGC	*Pst*I
gfpF	ATAAAAAATCGAAAAGGAGATAAAAAGATATGAGCAAAGGAG	
gfpR	CGCGAATTCTTAGTAGAGCTCATC	*Eco*RI

^a^The restriction sites in the primer sequences are underlined

### Bioinformatic analysis

A multiple-sequence alignment was performed using Clustal X, version 2.0 [30] and ESPript 3.0 [31]. The amino acid sequences of PheS proteins from six LAB strains were aligned with the amino acid sequences of PheS proteins from *E. coli* [27] and *E. faecalis* [32].

### Construction of the counterselectable system PheS*/pG^+^host9 in *Lc. lactis*

The counterselectable P_ldh_-pheS* cassette was constructed using an overlap extension PCR strategy. The constitutive promoter region of the l-lactate dehydrogenase gene (*ldh*) (accession number: NC_017949) in *Lc. lactis* NZ9000 [33] was amplified by PCR using primer pair ldhF1 and ldhR1. The pheS* gene was generated as two fragments by PCR using the *Lc. lactis* NZ9000 chromosomal DNA as a template with the primer pairs pheSF and siteR, siteF and pheSR, respectively. The point mutation responsible for *p*-Cl-Phe sensitivity was introduced by the primers siteF and siteR annealing internal to the wild-type *pheS* gene. There are overlapping regions among the three amplicons, which allowed an overlap extension PCR step using primers ldhF1 and pheSR to create P_ldh_-pheS* cassette. The generated 1270 bp P_ldh_-pheS* cassette was digested with *Eco*RI and *Bgl*II and ligated to the compatible sites of *Lc. lactis*/*E. coli* shuttle vector pSec:Leiss:Nuc [34], creating pleiss-P-pheS*.

To investigate the feasibility of the counterselectable P_ldh_-pheS* cassette, the plasmid pleiss-P-pheS* was introduced into the competent cells of *Lc. lactis* NZ9000 by electroporation [35]. The recombinant strain *Lc. lactis* NZ9000/pleiss-P-pheS* was incubated in GM17 with 5 µg/ml chloramphenicol. Overnight cultures were tenfold serially diluted, and 5 µl of diluted solution were pipetted onto air dried GM9 plates containing 0, 5, 10, 15 mM *p*-Cl-Phe, the cell survival was measured.

### Construction of plasmids pleiss-nP-gfp

To demonstrate whether cascading promoters could increase the *gfp* gene expression, a series of plasmids pleiss-nP-gfp carrying promoter clusters nP_ldh_-*gfp* were constructed as follows: the *gfp* gene was PCR amplified using primers gfpF and gfpR from plasmid pOgfp [18]. the promoter P_ldh_ and *gfp* gene were fused by an overlap extension PCR using primers ldhF2 and gfpR. The resulting product P_ldh_-*gfp* was digested with *Bgl*II and *Eco*RI and ligated into the corresponding sites of pSec:Leiss:Nuc [34] to create plasmid pleiss-P-gfp. The promoter P_ldh_ was generated by PCR with primers ldhF2 and ldhR2, and the PCR product was digested with *Bgl*II and *Bam*HI and inserted into the *Bgl*II site of pleiss-P-gfp to generate pleiss-2P-gfp. The same procedure was carried out to construct the plasmid pleiss-nP-gfp (n: the copy number of P_ldh_ in the promoter clusters nP_ldh_-*gfp*). Then, the above plasmids pleiss-nP-gfp were introduced into *Lc. lactis* NZ9000.

### Fluorescence assay

Recombinant strains harboring the pleiss-nP-gfp were grown aerobically in 5 ml GM17 broth containing 5 µg/ml chloramphenicol at 30 °C. Samples for measurement were taken out after 12 h and harvested by centrifugation at 10,000×*g* for 3 min. After being resuspended twice with PBS buffer (137 mM NaCl, 2.7 mM KCl, 10 mM Na_2_HPO_4_, 2 mM KH_2_PO_4_, pH7.4), 200 μl of bacterial suspension was transferred into a 96-well plate in which OD_600_ and fluorescence were read with excitation at 485 nm and emission at 528 nm using a Multi-Detection Microplate Reader, Synergy HT (BioTek). For each sample, three repetitions were performed with PBS buffer as a blank.

### Construction of counterselectable cassettes nP_ldh_-pheS* and plasmids pG^+^-nP-pheS*

To increase the expression of PheS* protein, a series of counterselectable cassettes, namely nP_ldh_-pheS* were constructed as follows: the P_ldh_-pheS* cassette was PCR amplified using primer pair ldhF3 and pheSR2 from plasmid pleiss-P-pheS*. The resulting DNA fragment was digested with *Sal*I and *Eco*RI and ligated into the corresponding sites of pUC19 to create plasmid pUC-P-pheS*. The promoter P_ldh_ was generated by PCR with primers ldhF3 and ldhR3, and the PCR product was digested with *Xho*I and *Sal*I and inserted into the *Sal*I site of pUC-P-pheS* to generate pUC-2P-pheS. The same procedure was carried out to construct the plasmid pUC-nP-pheS (n: the copy number of P_ldh_ in the nP_ldh_-pheS* cassettes). To develop a counterselectable system in *Lc. lactis*, the above plasmids pUC-nP-pheS* were digested with *Sal*I and *Eco*RI, and the generating nP_ldh_-pheS* cassettes (Fig. 4a) were ligated to the *Sal*I and *Eco*RI sites of pG^+^host9 to yield plasmid pG^+^-nP-pheS*.

### Functional analysis of the pG^+^-nP-pheS* in *Lc. lactis*

To knockout the 709 bp fragment of the galactose operon in *Lc. lactis* NZ9000, pG^+^UD-nP-pheS* was constructed as follows. Upstream with 1000 bp in size (amplified with primers upF and upR) and downstream with 1011 bp (amplified with primers downF and downR) homology arms were PCR amplified from the genomic DNA of *Lc. lactis* NZ9000 and spliced by an overlap extension PCR using primers upF and downR; Subsequently, the fused fragment was digested by *Kpn*I and *Xho*I and inserted into the corresponding sites of the temperature-sensitive vectors pG^+^host9 [16] and pG^+^-nP-pheS*, resulting the plasmids pG^+^UD and pG^+^UD-nP-pheS*, respectively. The plasmid pG^+^UD and pG^+^UD-nP-pheS* were introduced into *Lc. lactis* NZ9000 to perform double-crossover homologous recombination as described previously [16]. Briefly, the recombinants were grown at 28 °C until OD_600_ 0.8–1.0, then transferred to 37 °C for 2 h to allow the single-crossover integrants growth. Appropriate cultures were plated onto GM17 medium with 5 µg/ml erythromycin at 37 °C. Subsequently, the single-crossover integrants were cultured in GM17 medium without erythromycin at 28 °C for excision of the vector by a second crossover process. The cultures were then plated onto GM9 plates containing 15 mM *p*-Cl-Phe at 37 °C. The single-crossover integrants and double-crossover mutants were both verified utilizing primer pair testF and testR.

To further confirm the function of pG^+^-5P-pheS*, the *aldB* gene encoding α-acetolactate decarboxylase was knocked out from the *Lc. lactis* NZ9000 genome using the above protocols. Primer pairs of aldB-uF/aldB-uR and aldB-dF/aldB-dR were utilized for amplifying the upstream and downstream homology arms, and fused by an overlap extension PCR. The resultant ~2.0 kb fragment was digested and inserted into the *Kpn*I and *Xho*I sites of the vector pG^+^-5P-pheS*. Subsequently, the yielding pG^+^UD2-5P-pheS* was transferred into *Lc. lactis* NZ9000 to perform the double-crossover homologous recombination as described above. The mutant *Lc. lactis* dA was verified by PCR with the primer pair aldB-testF and aldB-testR, and the mutant genotype was also confirmed by sequence analysis (Biosune Company, Shanghai, China).

### Extending this counterselectable marker pheS* to other LAB

To extend this counterselectable marker pheS* to other LAB, *L. casei* was selected as a host. The mutant gene pheS* was generated as two fragments by an overlap extension PCR using the *L. casei* BL23 [36] genomic DNA as a template with the primers BL-pheSF and BL-siteR, BL-pheSR and BL-siteF, respectively. The point mutation responsible for *p*-Cl-Phe sensitivity was PCR amplified by the primer pair BL-siteF and BL-siteR annealing internal to *pheS* gene. The generated pheS* was digested with *Pst*I and *Bgl*II and ligated to the compatible sites of pTRKH2 [37], creating pTRKH2-pheS*.

To investigate the feasibility of the counterselectable marker *p* heS*, the plasmid pTRKH2-pheS* was introduced into *L. casei* BL23 by electroporation [13]. The recombinant *L. casei* BL23/pTRKH2-pheS* was incubated into MRS with 5 µg/ml erythromycin. Overnight cultures were streaked onto a GM9 plate containing 10 mM *p*-Cl-Phe, the cell survival was measured.

## Results

### Bioinformatic analysis of PheS protein in selected LAB species

**Fig. 1:**
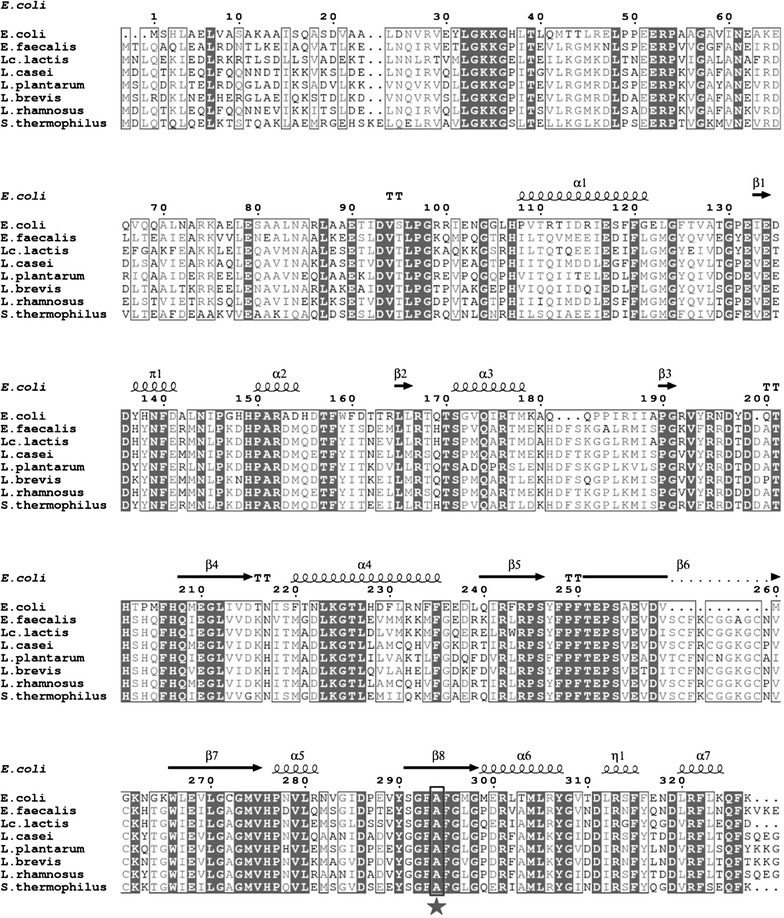
A multiple-sequence alignment of PheS from a variety of distantly related species. Full length sequences of PheS were determined using Clustal X. The secondary structure of PheS in *E. coli* (PDB code: 3PCO) is shown at the *top* of each set of sequence. The conserved alanine residues mutated to generate *p*-Cl-Phe sensitivity were boxed with a *thick line* and indicated with a pentagram. *α* α-helix, *β* β-sheet, *π* π-helix, *η* 3_10_-helix, *TT* β-turn

Previously, it was reported that only a point mutant pheS* gene encoding an A294G substitution in *E. coli* PheS [27] or an A312G substitution in *Enterococcus faecalis* PheS [32] resulted in the obviously sensitivity to the phenylalanine analog *p*-Cl-Phe. Hence, to identify the amino acid residue for site-directed mutagenesis, the amino acid sequences of PheS from *Lc. lactis*, *L. casei*, *L. plantarum*, *L. brevis*, *L. rhamnosus* and *S. thermophilus* were aligned with the amino acid sequences from *E. coli* and *E. faecalis* by Clustal X version 2.0 [30] and ESPript 3.0 [31]. As shown in Fig. 1, the amino acid residues A312 in *Lc. lactis* NZ9000, A312 in *L. casei*, A312 in *L. plantarum*, A312 in *L. brevis*, A312 in *L. rhamnosus* and A314 in *S. thermophilus* are strictly conserved compared to the residue A294 in *E. coli* [27] and A312 in *E. faecalis* [32], indicating that this alanine residue of PheS protein was highly conserved in LAB.

To verify the above putative result, we chose the amino acid residues A312 in *Lc. lactis* NZ9000 PheS for site-directed mutagenesis. The *pheS* gene (accession number: NC_009004) encoding phenylalanyl-tRNA synthetase alpha subunit was identified from the genome of *Lc. lactis* NZ9000. It was 1038 bp in size and composed of 345 amino acid residues. After precision mutation GCT to GGT by an overlap extension PCR strategy in codon 312 of the *pheS* gene, an A312G point mutation was introduced into the PheS protein, resulting a dominant-negative mutant protein PheS*. Moreover, we also tested the potential of A312G point mutation of PheS for counterselection in *L. casei* BL23.

### Functional analysis of the counterselectable marker P_ldh_-pheS* in *Lc. lactis*

**Fig. 2:**
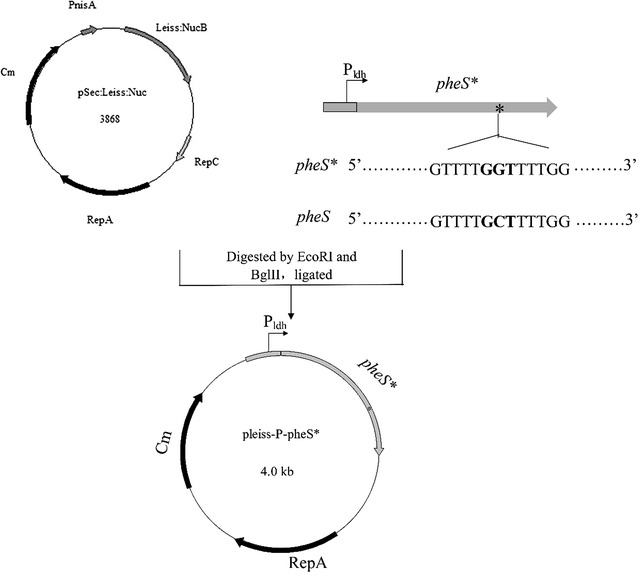
Construction of a vector for detecting the PheS*/pG^+^host9 counterselectable system. The wild-type *pheS* was changed to pheS* using an overlap extension PCR to introduce a point mutation of GCT to GGT. P_ldh_: the promoter region of the l-lactate dehydrogenase gene (*ldh*) (accession number: NC_017949) from *Lc. lactis* NZ9000

To test the feasibility of the gene pheS* as a counterselectable marker in *Lc. lactis*, it was driven constitutively by a strong promoter of l-lactate dehydrogenase gene (*ldh*) in *Lc. lactis* NZ9000. Subsequently, this resultant P_ldh_-pheS* cassette was inserted into the *Lc. lactis*/*E. coli* shuttle vector pSec:Leiss:Nuc, yielding the recombinant plasmid pleiss-P-pheS*. The schedule of construction of pleiss-P-pheS* was shown in Fig. 2.

**Fig. 3:**
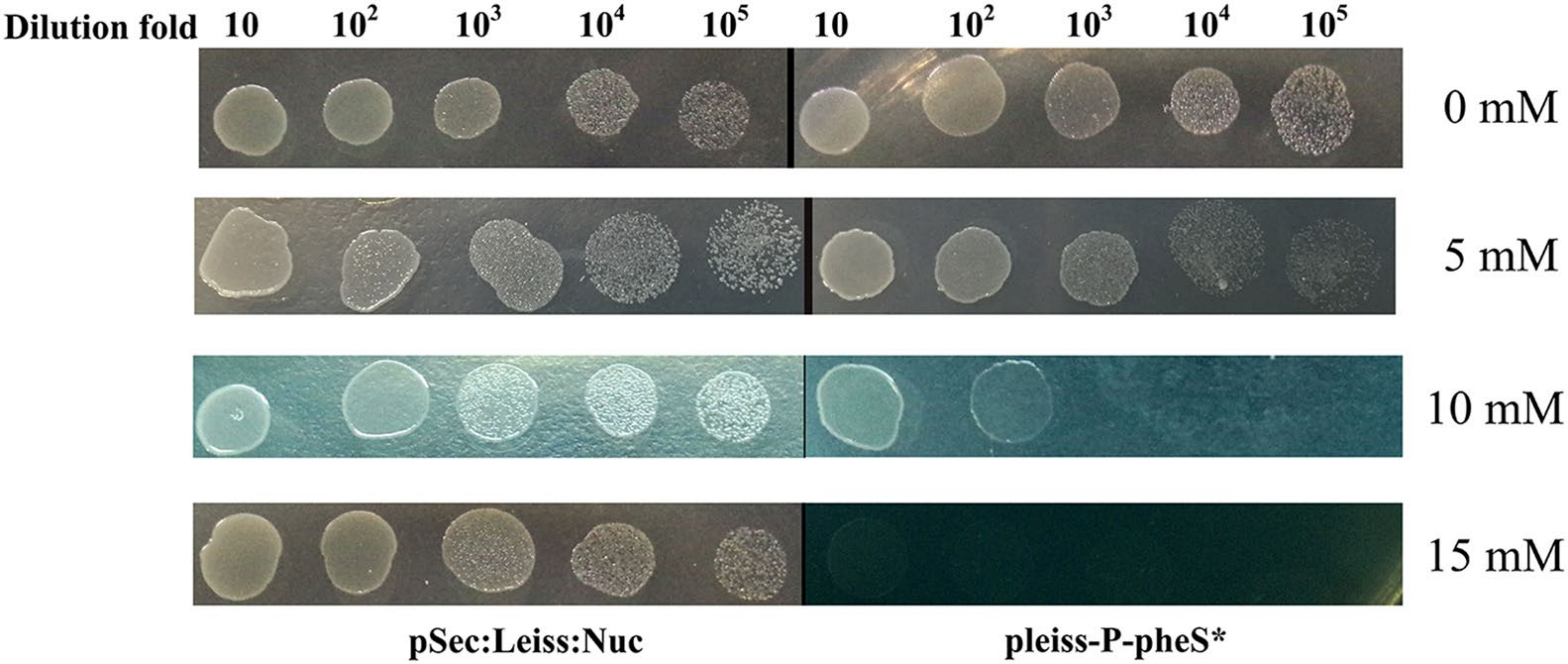
Detection of the sensitivity of *Lc. lactis* NZ9000/pleiss-P-pheS* to *p*-Cl-Phe. Wild-type *Lc. lactis* NZ900 carrying either an empty vector (pSec:Leiss:Nuc) or a *Lc. lactis* derivative carrying the P_ldh_-pheS* cassette (pleiss-P-pheS*) were cultivated overnight in GM9 plates containing the indicated concentration of *p*-Cl-Phe. This experiment was performed in triplicate with the similar results

After introduction of the plasmid pleiss-P-pheS* into *Lc. lactis* NZ9000, the sensitivity of the recombinant *Lc. lactis* NZ9000/pleiss-P-pheS* was measured on the GM9 plates containing 0, 5, 10, 15 mM phenylalanine analog *p*-Cl-Phe, respectively. As shown in Fig. 3, *Lc. lactis* NZ9000/pleiss-P-pheS* grew well on the GM9 plate without *p*-Cl-Phe, while the growth was completely inhibited in the presence of 15 mM of *p*-Cl-Phe. But, unlike the *Lc. lactis* NZ9000/pleiss-P-pheS*, the growth of *Lc. lactis* NZ9000 equipped with the plasmid pSec:Leiss:Nuc as a control was not inhibited under the equivalent concentrations of *p*-Cl-Phe, indicating that the dominant-negative mutant protein PheS* is functional as a stringent counterselectable marker in the presence of 15 mM *p*-Cl-Phe.

### Creation of a counterselectable cassette 5P_ldh_-pheS* in *Lc. lactis*

The temperature sensitive plasmid pG^+^host9 was widely used for gene(s) deletion and insertion in LAB. Here, a counterselectable system PheS*/pG^+^host9 was constructed based on a pG^+^host9 carrying the fragment P_ldh_-pheS* from the plasmid pleiss-P-pheS*, and yielding pG^+^-P-pheS*. To investigate the feasibility of this vector for gene deletion, upstream and downstream homology arms of the 709 bp fragment of galactose operon were spliced and inserted into pG^+^host9 and pG^+^-P-pheS*, resulting pG^+^UD and pG^+^UD-P-pheS*. The single-crossover integrants *Lc. lactis* IG0 and *Lc. lactis* IG1 were both pipetted onto GM9 plates containing 15 mM *p*-Cl-Phe, respectively. Unfortunately, *Lc. lactis* IG1 was not completely inhibited by *p*-Cl-Phe. We supposed that this unexpected phenomenon might resulted from the low expression of PheS* protein from a chromosomal copy.

**Fig. 4:**
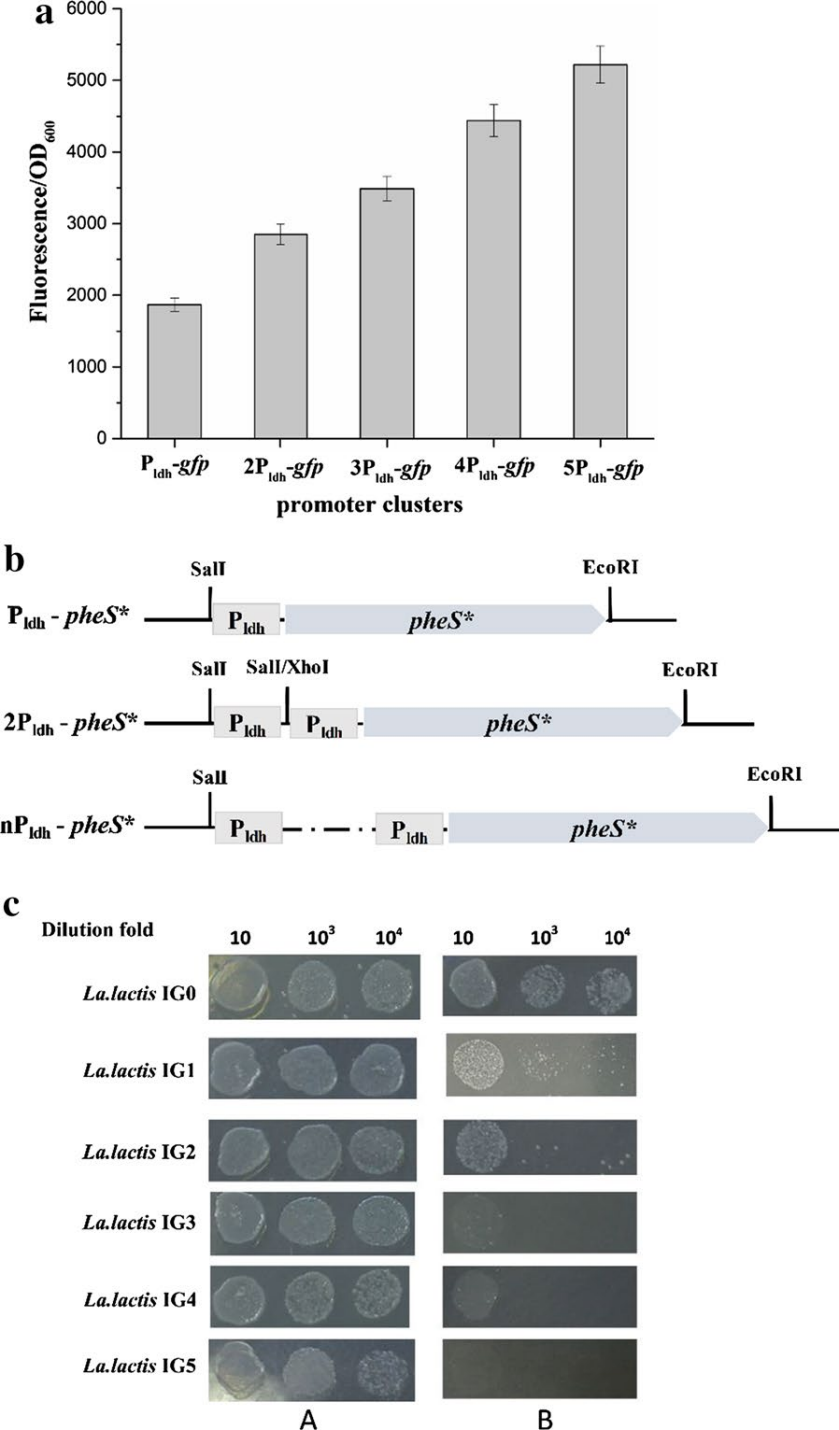
Assessment of the counterselection with nP_ldh_-pheS* cassettes in *Lc. lactis* NZ9000. **a** Determination of the transcription strength of the nP_ldh_-*gfp* promoter clusters by fluorescence analysis. **b** Structure of nP_ldh_-pheS* cassettes used in this study. **c** Detection of the sensitivity of various integrants to 15 mM *p*-Cl-Phe. *Lc. lactis* IGn indicates a derivative of *Lc. lactis* NZ9000 in which the nP_ldh_-pheS* cassette was inserted into the genomic DNA by a single-crossover process. *A* GM9 plates; *B* GM9 plates with 15 mM *p*-Cl-Phe. The *letter* of “n” means the copy number of P_ldh_

To increase the expression level of pheS*, we firstly tested whether cascading promoters could be functional in *Lc. lactis*. A series of plasmids pleiss-nP-gfp carrying promoter clusters nP_ldh_-*gfp* were constructed and introduced into the *Lc. lactis* NZ9000. To optimize the constructed nP_ldh_-*gfp* promoter clusters, fluorescence intensity of each construct radiated from the green fluorescence protein after 12 h of aerobic incubation was determined. By analyzing the cell growth and relevant fluorescence of each recombinant strain, we found that the more copies of the P_ldh_ promoter were present in the expression cassette, the higher the specific fluorescence was (Fig. 4a). This result indicated that cascading promoters could improve the expression level of *gfp* gene.

Subsequently, various copies of the P_ldh_ were driven the expression of dominant-negative mutant protein PheS* (Fig. 4b). As shown in Fig. 4c, the increase of the P_ldh_ copies in the nP_ldh_-pheS* cassettes inserted into the chromosomal locus of the integrants resulted in the enhanced sensitivity to 15 mM *p*-Cl-Phe. When the pheS* gene expressed from five copies of the P_ldh_, the growth of *Lc. lactis* IG5 was substantially inhibited. Thus, we chose the 5P_ldh_-pheS* cassette as a negative selectable marker for development of a counterselectable seamless mutagenesis system PheS*/pG^+^host9 in *Lc. lactis*.

### Functional analysis of the counterselectable system PheS*/pG^+^host9 in *Lc. lactis*

**Fig. 5:**
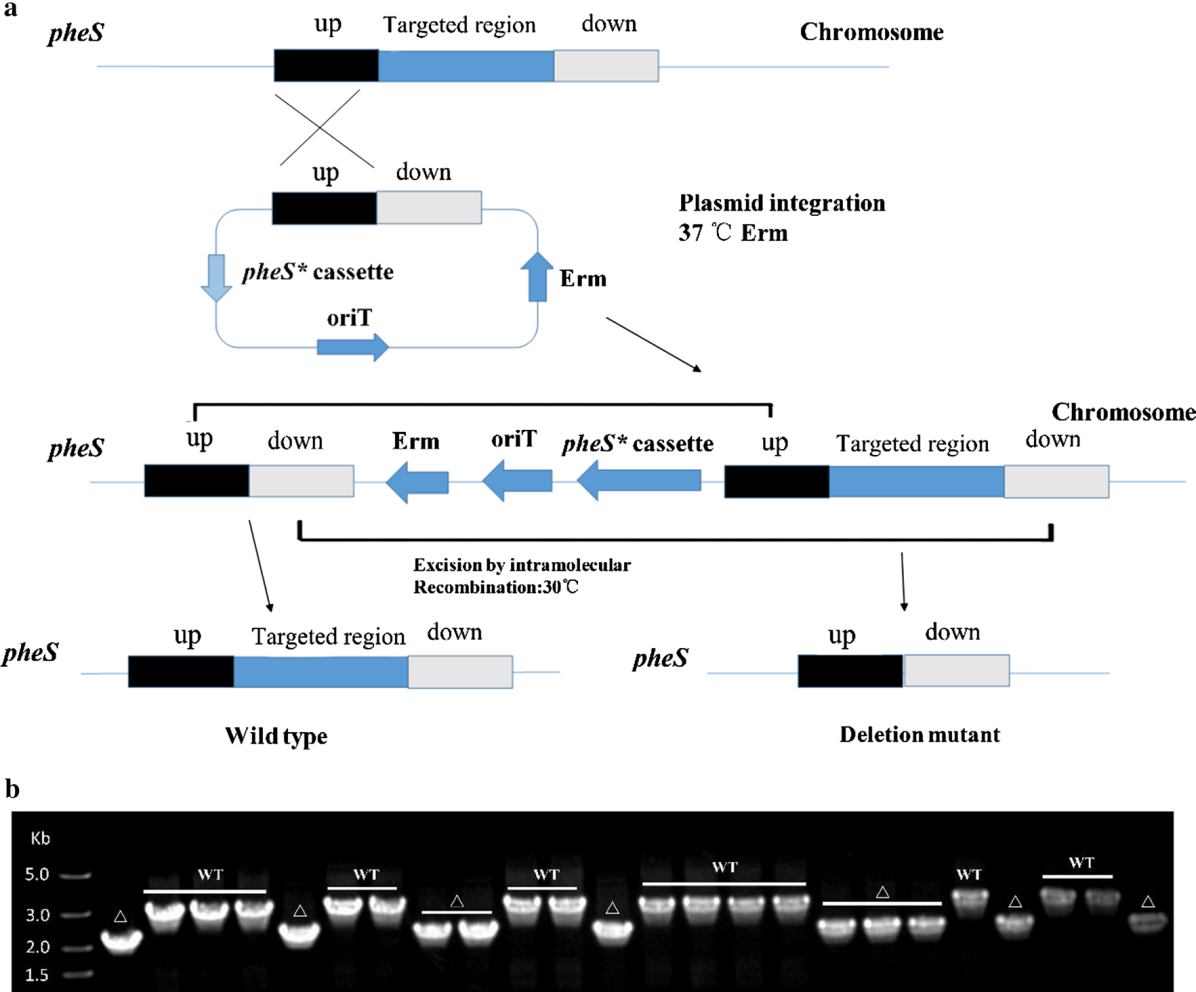
Construction of the PheS*/pG^+^host9 counterselectable system in *Lc. lactis* NZ9000. **a** An effcient counterselectable system PheS*/pG^+^host9 used to create gene deletions in *Lc. lactis* NZ9000. The “pheS* cassette” indicates the pheS* gene under the control of five cascading P_ldh_. “*up*” and “*down*” indicate the upstream and downstream homology arms of the targeted region. “Erm” indicates the erythromycin resistant gene. “OriT” indicates the temperature sensitive origin of replication. **b** Twenty-four *p*-Cl-Phe-resistant colonies were amplified by PCR to screen for the deletion of 709 bp fragment of the galactose operon. The expected PCR fragment from the mutant type (∆) is approximately 2.0 kb, while the band from the wild-type (WT) is about 2.7 kb

To verify the potential of the counterselectable system PheS*/pG^+^host9 in *Lc. lactis* NZ9000, a 709 bp fragment of the galactose operon was selected as a targeting region for deletion through the vector pG^+^UD-5P-pheS*. After *p*-Cl-Phe counterselection, 24 resistance colonies picked randomly on a GM9 plate containing 15 mM *p*-Cl-Phe were all double-crossover occurred and 10 out of them (approximately 42%) were shown to possess the expected mutant genotype by PCR determination and sequencing. The result was closed to the theoretical value (50%) since the target region of the galactose operon was not essential for the growth of *Lc. lactis* NZ9000 (Fig. 5a, b). Hence, we supposed that the 5P_ldh_-pheS* cassette based counterselectable system was functional in *Lc. lactis* NZ9000 to perform seamless gene deletion.

**Fig. 6:**
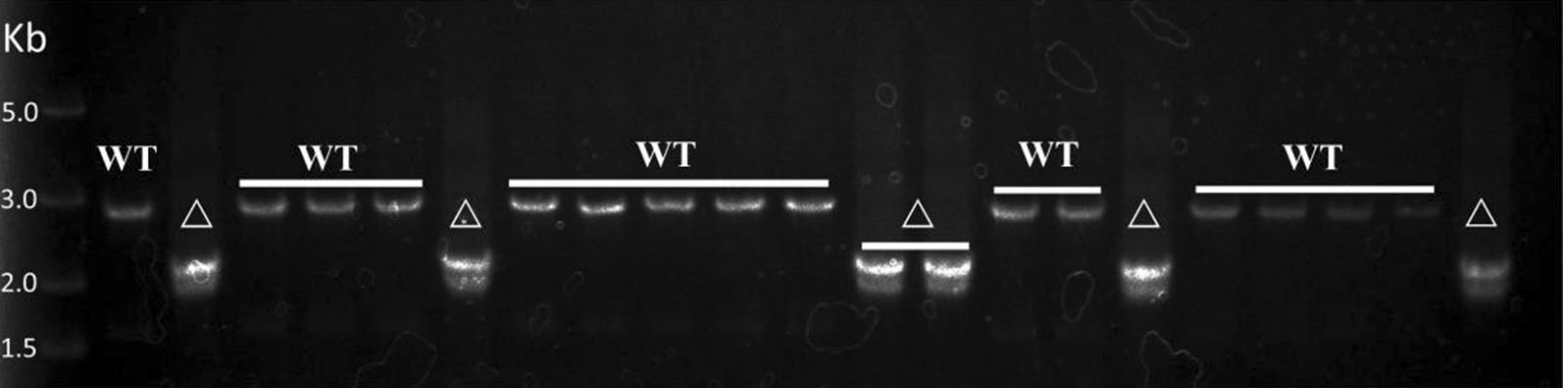
Generation of a seamless in-frame *aldB* deletion mutant. Twenty-one *p*-Cl-Phe-resistant colonies were amplified by PCR to screen for the deletion of the *aldB* gene (711 bp). The expected PCR fragment from the mutant type (∆) is approximately 2.0 kb, while the band from the wild-type (WT) is about 2.7 kb

To further determine whether the counterselectable system PheS*/pG^+^host9 would be feasible for genome engineering, *aldB* gene which encodes for α-acetolactate decarboxylase catalyzing α-acetolactate to acetoin in the diacetyl biosynthesis in *Lc. lactis* was deleted by this system (Fig. 5a). Twenty-one colonies were selected randomly and detected by PCR amplification. The double-crossover events also occurred in 100%, and six out of them were the expected mutants (Fig. 6). This result indicated that the efficiency of screening double-crossover mutants was significantly improved compared with using pG^+^host9 alone in our laboratory previously [18].

### Potential of the counterselectable marker pheS* in other LAB

**Fig. 7:**
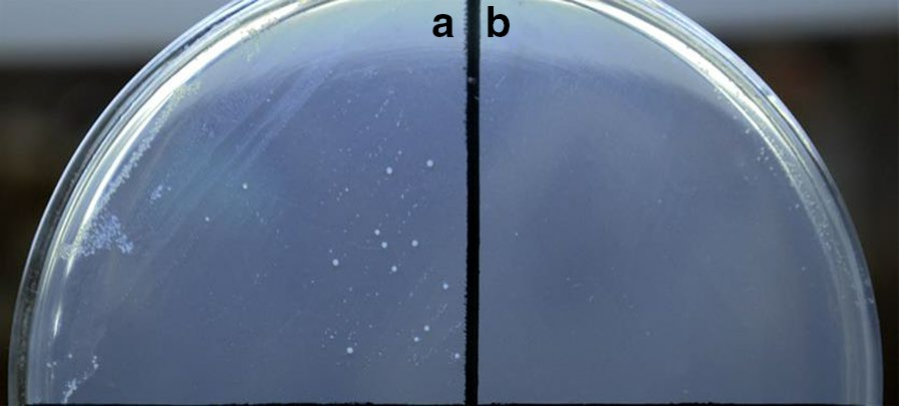
*p*-Cl-Phe sensitivity of *L. casei* strains. Wild-type *L. casei* BL23 carrying an empty shuttle vector (**a**) or a derivative carrying the pheS* (**b**) were cultivated overnight in GM9 plates containing 10 mM *p*-Cl-Phe at 37 °C

To test the feasibility of the gene pheS* as a counterselection marker in other LAB, the strain *L. casei* BL23 was selected as a host. After insertion of pheS* into pTRKH2 [37], the obtained plasmid pTRKH2-pheS* was introduced into *L. casei* BL23. Subsequently the sensitivity of the recombinant *L. casei* BL23/pTRKH2-pheS* to *p*-Cl-Phe was measured on the GM9 plates containing 10 mM *p*-Cl-Phe. As shown in Fig. 7, the recombinant *L. casei* BL23/pTRKH2 grew well on the GM9 plate containing 10 mM *p*-Cl-Phe, while the growth of *L. casei* BL23/pTRKH2-pheS* was obviously inhibited, indicating that the conditional-lethal mutant gene pheS* has the potential as a counterselectable marker in *L. casei* and other LAB.

## Discussion

In consideration of the increasing use in industrial and medical area, LAB are intensively studied on their genetics and metabolism [9, 10]. Therefore, efficient genome engineering tools are necessary for target gene(s) deletion or insertion for functional analysis or rerouting the metabolic flux [38]. In this study, a seamless negative selectable mutagenesis system PheS*/pG^+^host9 was developed. We also demonstrated its feasibility by constructing strains bearing the targeting seamless deletion of a 709 bp fragment in lactococcal galactose operon and *aldB* gene. Expectedly, the ratio of the double-crossover event was 100% after counterselection by *p*-Cl-Phe.

To our knowledge, this is the first report that the mutated *pheS* allele can be used as a counterselection marker for efficient and rapid genomic engineering in *Lc. lactis*. Previously, the development of a *pheS* based counterselection system in *Streptococcus mutans*, which is a close relative to *Lc. lactis*, has been reported [28]. However, *S. mutans* is a pathogenic bacterium distributed in the dental caries and could not be applied in the food field and used as a cell factory [39]. We expected that combining the counterselectable marker pheS* with the traditional genetic tool pG^+^host9 [16] would overcome the bottleneck of laboriously screening of the double-crossover recombinants, and this system has greatly potential in genome engineering in LAB.

Protein sequence analysis suggested that the alanine residue of PheS protein is highly conserved in LAB (Fig. 1). Here we have demonstrated the feasibility of pheS* as a counterselectable marker in *Lc. lactic* and *L. casei*, these results were consistent with the previously results in *S. mutans* and *Enterococcus faecalis* [28, 32]. Therefore, we speculated that the dominant-negative mutant gene pheS* might be widely used as a counterselectable marker in a variety of lactic acid bacterial species. However, the sensitivity of the cells to *p*-Cl-Phe was depended on strain specific manner, such as 15 mM *p*-Cl-Phe for *Lc. lactis* NZ9000, 20 mM *p*-Cl-Phe for *S. mutans* [28]. Hence, optimization of the PheS* expression is needed when employing pheS* as a counterselectable marker in other LAB strains [25, 28].

In this study, the PheS* protein under the control of a promoter P_ldh_ has the ability of completely inhibiting the growth of *Lc. lactis* NZ9000 at 15 mM *p*-Cl-Phe, suggesting it is possible to use P_ldh_-pheS*cassette as a counterselectable marker in *Lc. lactis*. However, the growth of the recombinants with P_ldh_-pheS* inserted into the chromosomal locus was not completely inhibited by even higher concentration of *p*-Cl-Phe. This unexpected result means that the ratio of screening double-crossover recombinants would not be 100% after *p*-Cl-Phe counterselection. We speculated that this phenomenon was caused by low expression level of PheS* [28], because the copy number of P_ldh_-pheS* from the chromosomal locus was lower than that in the plasmid pleiss-P-pheS*. Lower yield of PheS* was insufficient to compete with the background expression of wild-type PheS to form complexes with phenylalanyl-tRNA synthetase beta subunit (PheT) [40]. In these cases, a strategy of cascading promoters [41] was employed to improve the expression level of protein PheS*. Surprisingly, when protein PheS* was driven simultaneously by five copies of the P_ldh_, the generating integrant *Lc. lactis* IG5 was substantially inhibited in the presence of 15 mM *p*-Cl-Phe and the ratio of screening double-crossover recombinants was 100%, suggesting recombination among the promoters was not occurred and the use of repeated P_ldh_ promoters could not confer genetic instability [41]. This strategy provides a new idea to address the issue of the low expression of the exogenous protein(s) in LAB.

Several strategies have been employed to fulfill the genome engineering in LAB by homologous double-crossover using a solely conditional replication plasmid [38] or combining with other counterselectable system, such as *upp* [22] or *oroP* [24] based cassettes. Compared with those methods, the negative selectable system PheS*/pG^+^host9 has several advantages. (1) It greatly simplifies the procedure for screening double-crossover recombinants. For example, taking only 2 days to screen double-crossover variants after the single-crossover integrants were subcultured at 28 °C. The ratio of the double-crossover recombinants was 100% after *p*-Cl-Phe counterselection. However, the ratio between the deletion and wild-type strains may not be the theoretical value (1:1), it can vary considerably depending on the function of gene(s) to be deleted. (2) To our knowledge, among all the reported counterselectable markers, only pheS* has the greatly potential to be widely utilized in wild-type LAB without pretreatment. In contrast to other counterselectable system, the variants required the counterselectable marker deficient strains, as in the case of *upp* [21–23] and *oroP* [24]. Recently, a new counterselection method for wild-type *Lc. lactis* genome engineering based on class IIa bacteriocin sensitivity was reported [42]. However, the li006Dlitation of this method to be widely used in LAB was the sensitivity to bacteriocins which would depend on the interaction between the listerial MpnC and the native PtnD [42]. (3) Strains without pheS* can naturally grow on GM9 medium with 15 mM *p*-Cl-Phe. This means 15 mM *p*-Cl-Phe has no side-effect on the growth of the expected mutants.

Moreover, this mutagenesis system PheS*/pG^+^host9 allowed gene deletion without any genomic scarring [15] in *Lc. lactis*, as the case of the *aldB* gene. The generating genetically modified microorganisms (GMOs) [14] were seamless mutagenesis which means only leaving self-DNA in its native genome location [15]. Therefore, this system is useful in seamless gene deletions in industrial strains. However, this seamless mutagenesis system PheS*/pG^+^host9 remains challenging in large DNA fragment deletions or insertions. In this study, the limited length of the targeted DNA fragment was mostly from the low efficient homologous recombination mediated by RecA [17]. In consideration of the ratio of the double-crossover recombinants was 100% after *p*-Cl-Phe counterselection, the ideal goal for deletion or insertion of large DNA fragment is the new genome engineering tools responsible for targeted fragments replacement by selection and the 5P_ldh_-pheS* cassette responsible for selectable marker excision by counterselection [15].

## Conclusions

A seamless mutagenesis system PheS*/pG^+^host9 based on a counterselectable marker pheS* and a temperature sensitive plasmid pG^+^host9 was developed in *Lc. lactis*. Moreover, this system can be used for rapidly constructing a seamless mutagenesis (deleted or inserted) strain. We also extended the counterselectable marker pheS* to *L. casei*. Although the feasibility of pheS* as a counterselectable marker used in other LAB remains to be demonstrated, we speculated that this conterselectable marker will accelerate the analysis of genes with unknown function and metabolic engineering research in LAB.

## Abbreviations

LAB: lactic acid bacteria
*pheS*: gene of phenylalanyl-tRNA synthetase alpha subunit
*pheS**: the mutant *pheS* gene
PheS*: phenylalanyl-tRNA synthetase alpha subunit with an A312G substitution
P_ldh_: promoter of the *Lc. lactis* NZ9000 L-lactate dehydrogenase
*p*-Cl-Phe: *p*-chloro-phenylalanine
SSDR: single-stranded DNA recombineering
DSDR: double-stranded DNA recombineering
